# Phonetic Imitation from an Individual-Difference Perspective: Subjective Attitude, Personality and “Autistic” Traits

**DOI:** 10.1371/journal.pone.0074746

**Published:** 2013-09-30

**Authors:** Alan C. L. Yu, Carissa Abrego-Collier, Morgan Sonderegger

**Affiliations:** 1 Phonology Laboratory, Department of Linguistics, University of Chicago, Chicago, Illinois, United States of America; 2 Department of Linguistics, McGill University, Montreal, Quebec, Canada; UNLV, United States of America

## Abstract

Numerous studies have documented the phenomenon of phonetic imitation: the process by which the production patterns of an individual become more similar on some phonetic or acoustic dimension to those of her interlocutor. Though social factors have been suggested as a motivator for imitation, few studies has established a tight connection between language-external factors and a speaker’s likelihood to imitate. The present study investigated the phenomenon of phonetic imitation using a within-subject design embedded in an individual-differences framework. Participants were administered a phonetic imitation task, which included two speech production tasks separated by a perceptual learning task, and a battery of measures assessing traits associated with Autism-Spectrum Condition, working memory, and personality. To examine the effects of subjective attitude on phonetic imitation, participants were randomly assigned to four experimental conditions, where the perceived sexual orientation of the narrator (homosexual vs. heterosexual) and the outcome (positive vs. negative) of the story depicted in the exposure materials differed. The extent of phonetic imitation by an individual is significantly modulated by the story outcome, as well as by the participant’s subjective attitude toward the model talker, the participant’s personality trait of openness and the autistic-like trait associated with attention switching.

## Introduction

Imitation has been observed in many domains of human behavior, including postures, gestures, and facial expressions [9]. In the domain of language and speech, imitation has been observed for many properties, such as lexical and syntactic alignment [2], speech rate [3], pause and utterance duration [4], vocal intensity [5], vowel quality [6], [7], and voice onset time (VOT) [8]–[10]. When the speech production patterns of an individual become more similar on some phonetic or acoustic dimension to those of her interlocutor, phonetic *imitation* or *convergence* obtains; phonetic *divergence* refers to the reverse process. For example, studies using a “shadowing” paradigm (e.g., [11], [12]) show that subjects shift their speech production (evaluated using perceptual measures) in the direction of speech they are asked to repeat, either immediately or after a delay. Several previous studies have considered imitation of VOT in particular. A significant VOT imitation effect was reported in a single-word shadowing task using words with artificially-lengthened initial VOTs [13]. VOT imitation was also observed even when subjects were not explicitly asked to shadow: their VOTs became longer after listening to a period of speech with extended VOTs; subjects also generalized the extended VOT pattern to words they were not exposed to during the passive listening task [8], [9]. Given the prevalence of imitation effects in language, some scholars have hypothesized that studies of phonetic imitation and convergence can inform the understanding of sound change. In particular, phonetic imitation found in the laboratory setting is taken to be similar to phonetic convergence in conversational interaction, which has been hypothesized as an important source of propagation of sound changes throughout speech communities [7], [10], [14]–[16].

Phonetic imitation is not an entirely automatic (i.e., it can occur without the speaker’s intention or control) or unrestricted process [9]. Contrast preservation, which has been argued to be an essential part of the phonological grammar [17]–[19], may constrain phonetic imitation. For example, one study found that lengthened VOTs were imitated but shortened VOTs were not [1], suggesting that speakers may not imitate if the novel phonetic feature (shortened VOT) endangers phonetic contrasts (unaspirated vs. aspirated). In the case of vowels, subjects in one study imitated only low vowels but not higher ones [6], which might be due to the influence of speaker experience; unlike the higher vowels, subjects encounter more varieties of low vowels due to differences in jaw height in accented and unaccented syllables.

Beyond linguistic factors, “macro” socio-biological factors, such as gender/sex, have often been suggested as important moderators of imitation [1], [6], although the exact nature of this modulation is not clear. Men have been found to imitate more than women in a map task [14], but less than women in a shadowing task [20]. These mixed results suggest that gender/sex may not be the appropriate predictive factor in modulating likelihood of imitation.

Situational variables also affect the degree of imitation. Speakers vary in the degree of phonetic convergence depending on their gender as well as their role in a particular conversation. In a study where dyads participated in a map task, the degree of overall phonetic imitation (assessed perceptually) depended on the speaker’s conversational role, as well as gender [14], [16]. The language distance between interlocutors can also affect the likelihood of imitation; greater imitation is found in same-dialect conversational pairs than in either different-dialect pairs or different-L1 pairs [21].

Accommodation research, particularly work within the framework of Communication Accommodation Theory (CAT), which sees speech convergence phenomena as motivated by an individual’s desire for social acceptance and identification with a particular social group [22], has repeatedly demonstrated the centrality of subjective attitude and ideology for predicting the likelihood of linguistic convergence and divergence between speakers at multiple linguistic levels [23]–[25]. Many dialect convergence studies have found that speaker attitude and language ideologies strongly influence the degree of convergence between languages in contact. Labov, in his seminal study of/ay/and/aw/centralization in Martha’s Vineyard [26], showed that individuals who had a positive orientation toward the island were more likely to exhibit centralized diphthongs than those who did not. Another seminal study by Bourhis and Giles found that Welsh adults who were invested in Welsh language and culture would adopt a Welsh-accented dialect during the interview when talking to an out-group speaker, who questioned the vitality and function of the Welsh language in modern times [25]. On the other hand, Welsh adults who adopted a more utilitarian view of Welsh language and culture were more likely to accommodate to the interviewer. More recently, Babel found that speakers of New Zealand English were more likely to accommodate to an Australian talker in a speech production task when the New Zealand English speaker had a pro-Australia bias [27]. While these studies show that the extent of phonetic accommodation may be dependent on speaker attitudes, whether this type of attitude-based modulation of phonetic convergence would only arise from deep-seated attitudes formed over a long period or whether such modulation could be induced by impressions formed after short exposure (e.g., over the course of a conversation or over the course of a laboratory experiment) remains an open question. To the extent that subjects listening to a monologue may form an opinion of the narrator on account of attributes of the monologue, we hypothesize that listeners might show more accommodation to the narrator if the impression formed were positive. In addition to attitudinal differences, the effects of other individual-level factors on accommodation at the phonetic level have been investigated in recent years. Subjective evaluation of model talker attractiveness has been implicated as a potential modulating factor in vocalic imitation, as assessed using acoustic measures [6], [7]. “Phonetic talent” in L2 acquisition may also serve as a good predictor of phonetic convergence, as assessed by both perceptual and instrumental measures [28].

With the important exception of these studies, little is known about the factors which modulate interspeaker differences in the extent of phonetic imitation. Previous studies on phonetic imitation have largely focused on group-level effects, that is, effects observed in a (sub-)population as a whole. Understanding sources of individual differences in phonetic imitation is important for two reasons. While huge variability between speakers in the amount of phonetic imitation is often reported [7], [10], [14], there has been little discussion of what sources could underlie this variability. Given that sound change propagation hinges heavily on the attitude and stance of an individual within a locally-defined social reality, understanding why individuals differ in the extent of phonetic imitation is crucial for understanding the role of imitation phenomena in sound change. An individual’s personality profile and social distribution, for example, has been argued to play a significant role in contributing to the socially-structured distribution of linguistic innovations [29]–[32]. To the extent that personality traits and social dispositions are influenced, if only partially, by cognitive and neuropsychological factors [32], understanding the neuro-cognitive contribution to variation in phonetic imitation must also be seen as an integral part of unveiling the full picture of sound change actuation and propagation.

In this article, we aim to contribute to the existing literature on phonetic imitation by considering the range and relative magnitude of situational and individual-level factors that mediate phonetic imitation. To examine the effects of situational variables on phonetic convergence, we manipulate the nature of the model talker. Earlier studies have examined how phonetic imitation is affected by the conversational role of the participants [14], [16], the model talker’s perceived race/ethnicity [7], and speakers’ national identity and attitudes toward other countries [27]. In this study, we vary the talker’s perceived sexual orientation and the narrative’s outcome. The present investigation also considers the effects of individual-difference dimensions on phonetic imitation. As mentioned above, in addition to manipulating the nature of exposure materials, we consider the attitude of the listeners toward the narrator as a potential variable influencing phonetic imitation (see also [27]). Besides attitudinal differences, however, individuals also vary in terms of their intrinsic neuropsychological and cognitive predisposition. One important individual-difference dimension from this perspective is working memory capacity (WMC). WMC represents the ability to control attention and deal with irrelevant information, and not simply the amount of information that can reside in working memory [33]. The underlying premise of this controlled-attention viewpoint is that individual differences are not due to some limited amount of activation available to the working memory system but, rather, to an individual’s ability to ignore irrelevant information (on the basis of a specific relevant goal) through the control of attention [33], [34]. Individuals who possess lower WMC are generally less able to utilize executive control to ignore irrelevant or interfering information and maintain focus on a specific goal, whereas the opposite is true for high-WMC individuals. Availability of WM resources has been found to affect speech processing [35]. Increased WM load (thus reduced WM resources), for example, has been shown to slow down spoken word recognition [36]. High-WM individuals exhibit less perceptual compensation for coarticulation and are less biased toward hearing legal sound sequences than low-WM individuals [37]. WMC also affects sentence processing (see [38]) as well as success in learning artificial grammars [39], [40]. Thus to the extent that the type of perceptual retuning in phonetic imitation requires selective attention to the fine-grained phonetic details of the training materials, low-WM individuals might have more difficulty with phonetic imitation than high-WM ones.

Another individual-difference dimension is cognitive processing style and, by extension, personality traits. Cognitive processing style refers to psychological dimensions representing preferences and consistencies in an individual’s particular manner of cognitive functioning, with respect to acquiring and processing information [41]–[43]. Recent studies have suggested that across-individual variation in perceptual and production norms is determined in part by individual variability in cognitive processing style, such as traits associated with the Autism-Spectrum Condition (ASC), which includes autistic disorder, Asperger’s disorder, and pervasive developmental disorders, are characterized by deficits in social interaction, communication, and behavioral flexibility, and affects about 1% of the population. Autistic-like traits, as measured by the total Autism-Spectrum Quotient (AQ; [44]) taken from within the neurotypical population (i.e., individuals who are not clinically autistic), have been found to correlate negatively with the extent of identification shift associated with the `Ganong effect’ (i.e., the bias in categorization in the direction of a known word) [45]. Recent studies have also found significant associations between autistic-like traits and perceptual compensation for contextual variation in speech (vocalic context, [31]; talker voice [31]; and phonotactic contexts [37]), although the nature of the autistic-like trait effects varied depending on the type of contextual information. For example, individuals with high AQ exhibited stronger perceptual compensation for coarticulation than those with low AQ, while low-AQ individuals exhibited stronger phonotactic effects on speech perception than high-AQ ones. Individuals with ASC have been shown to exhibit enhanced perceptual processing of fine-grained auditory information [46], [47]. This suggests that individuals with more autistic-like trait-related cognitive processing styles might be particularly sensitive to fine phonetic differences. On the other hand, given that cognitive theories of autism hold that individuals with autism have difficulties integrating perceptual information with higher order language processing (weak central coherence; [48], [49]), neurotypical individuals with more pronounced autistic-like traits (e.g., high AQ), even if they might be better at detecting fine phonetic details, might nonetheless have difficulties utilizing the perceived fine phonetic differences in his/her own speech production to affect discernible phonetic imitation. Finally, variability in cognitive processing style has been shown to correlate with individual differences in social and personality traits. In particular, Autistic traits, as measured by the Autism-Spectrum Quotient and the Empathy Quotient [50], [51], has been shown to significantly correlated with individual personality traits [32], [52] and social network characteristics [32], [53]. Given that social and personality traits may influence how an individual interacts with other members of his/her social network or community of practice [29], [30], [32], we also gathered information regarding participants’ personality traits in an attempt to identify potential significant personality predictors of phonetic imitation.

## Materials and Methods

### Ethics Statement

The study was approved by the Social and Behavioral Sciences Institutional Review Board at the University of Chicago and written informed consent was obtained from all participants.

### Procedure

The production task consisted of three blocks: First, there was a *baseline* production block where subjects produced a list of 72/p t k/−initial target words (randomized order) in the carrier sentence, “say ___ again”. Target words, given in Table 1, were selected from celex2 [54], evenly distributed by token frequency quartile and by initial consonant. A subsequent post-exposure *test* block consisted of subjects producing the same word list again in a different randomized order. In between the two production tasks was a *exposure* block where subjects heard a constructed first-person narrative in which the same 72 p/t/k-initial words were embedded. VOTs of the target words in the story were extended by 100% using Praat [55]. VOTs were extended by selecting stable medial portions of the aspiration, copying, and pasting them back into the aspiration selection of the waveform (see also [13]). Intervals including sudden bursts of acoustic energy were avoided to minimize unnaturalness of aspiration noise. Care was also taken to select stretches of VOT that did not lead to perception of clicks or other evidence of the splicing operation when pasted back into the utterance. To achieve natural sounding extended VOT, duration and placement of selections varied across and within tokens.

**Table 1:** Stimuli for the baseline and test production blocks.

	Q1	Q2	Q3	Q4
Bilabial	picky	Pearl	patio	personal
	Pisces	pointlessness	pale	picture
	pensively	perfect	peppermints	put
	pink	purpose	panic-stricken	piece
	pimpled	peck	pain	pork
	poker-faced	pigsty	pair	pulse
Coronal	tingle	table	talker	tenth
	taunting	teasingly	tasteless	typical
	turn-on	temptingly	toffee	town
	teensy	tipsy	total	tolerable
	tearlessly	tigerish	tactful	terribly
	terror-stricken	tubby	timidly	two
Velar	cod	kinda	course	cocktail
	kissable	corpulent	cauliflower	contact
	chiropractor	captivate	candlelight	chemistry
	cusp	coaxing	candid	confidence
	concrete	cop-out	coolness	calm
	killjoy	cordial	courtship	compliment

Stimuli are arranged by frequency (by quartile in columns) and place of articulation.

All subjects took a post-experiment survey which included questions about the subject’s age, second language knowledge, assessment of own sexual orientation (from 1 = exclusively heterosexual to 7 = exclusively homosexual), feelings towards the talker (from 1 = very positive to 7 = very negative), likelihood of behaving in the same way in a similar situation (yes/no), and whether anything unusual was noticed in the talker’s speech. Subjects also completed several neurocognitive and personality measures. Subjects filled out the Autism-Spectrum Quotient (AQ; [44]). The AQ is a short, self-administered scale for identifying the degree to which any individual adult of normal IQ may have traits associated with the ASC, of which classic autism and Asperger’s Syndrome are the clearest subgroups. The AQ is not a diagnostic measure, although it has been clinically tested as a screening tool; traits as assessed by the AQ show high heritability and are stable cross-culturally. The test consists of 50 items, made up of 10 questions assessing five subscales: social skills (SS), communication (CM), attention to detail (AD), attention-switching (AS), and imagination (IM). The AQ items were scored on a Likert scale (1–4). A total AQ score was calculated by summing all the scores for each of the items, with a maximum score of 200 and a minimum score of 50. Scores for the subscales (AS, CM, AD, AS, IM) have a maximum score of 40 and a minimum score of 10. All scales were scored in such a way that high scores indicated traits associated with ASC: lower social skills, difficulty in attention switching/strong focus of attention, higher attention to detail and patterns, lower ability to communicate, and lower imagination. Subjects also took the Big Five Inventory, which consists of five broad personality dimensions: Openness (O), Conscientiousness (C), Extraversion (E), Agreeableness (A), and Neuroticism (N) [56], [57]. The score for each personality dimension was computed as the mean score for questions associated with the dimension. High AQ individuals are associated with high Neuroticism, low Extraversion, and low Agreeableness [32], [52] or low Conscientiousness [32], [58]. Subjects also completed the Automated Reading Span Task (RSPAN; [59]), a widely-used instrument for assessing working memory capacity. In this test, subjects were presented with a series of sentences on a computer (e.g., “The ranger told the hiker to look out for snakes.”) and were asked to indicate whether the sentence makes sense by clicking “TRUE” or “FALSE” on the screen. A letter was then presented for participants to hold in memory. These sentence-letter trials were presented in sets, with three to seven trials per set, for a total of 75 letters in 15 sets. At the end of each set, a screen with 12 letters appeared, and participants used the mouse to select the letters they remembered in the correct order. Scores for the RSPAN were calculated with the partial-credit unit scoring method [60]. The order in which the battery of personality/socio-cognitive tests was administered was random.

### Materials

The narrative consisted of a male talker recounting the experience of a recent blind date. The narrative contained no other stressed syllable-initial voiceless aspirated stops aside from the target words. Two versions of the narrative were created: in one version, the narrator abandoned his date at the restaurant and went home alone (the “negative” version); in the other version, the narrator hit it off with the date and was happy about it (the “positive” version). In order to manipulate the perceived sexual orientation of the narrator (as either “heterosexual” or “homosexual”), the gender of the date was varied for each storyline. A total of four possible storylines were created (i.e., two date outcomes (“positive” vs. “negative”) × two perceived narrator sexual orientations (“heterosexual” vs. “homosexual”)). The full texts of the heterosexual version of the “positive” and “negative” storylines are given in the Supporting Information S1. To create the narrative recording, a native English-speaking male talker was recorded reading all four versions of the story. The VOTs of the target words in the “homosexual” version of the narratives (both “positive” and “negative” outcomes) were extended as described above. The “heterosexual” version of the narratives was created by replacing the proper names and pronouns in the extended-VOT recordings with the gender-appropriate names and pronouns from the “heterosexual” versions of the recording.

### Measurements

A team of five labelers delineated and labeled target-word VOTs in Praat, using both waveforms and spectrograms to determine the extent of prevocalic aspiration. VOTs were then calculated using a script. Seven sets of test block recordings were measured by all five labelers and VOT measurements were compared in order to check for inter-researcher consistency. No single VOT had more than 6 msec of variation among the five measurements. Considering that the inter-researcher variation reported in previous VOT studies ranges from 2 msec to 10 msec [61]–[64], a difference in 6 msec of VOT variation appears to be reasonable. Moreover, given that our main focus is in the amount of within-individual VOT difference across blocks, variation in VOT across individuals is also less of a factor in the final analysis.

### Participants

Ninety-three subjects completed the study, and received either course credits or a nominal cash payment (USD$10). Participants were assigned to one of the four conditions. Approximately equal numbers of subjects participated in each of the conditions. (Note that our analysis below uses mixed-effects regression models, which are robust to unbalanced designs.).

## Results

While 93 subjects participated in the study, data from two subjects were lost due to problems with the recording procedure. In addition, data were excluded from one subject who said many words without the carrier phrase, as well as from six subjects who did not complete the RSPAN or one of the questionnaires (AQ or Big Five). While subjects read 72 words, due to problems with the stimuli presentation script, the words “pair” and “pearl” did not appear consistently across pre-exposure and post-exposure block and were excluded from the final analysis. The following analysis was performed on VOTs for the remaining 70 words by 84 subjects.

Descriptive statistics of subjects’ age, and attitude scores, as well as their AQ and Big Five scores are given in Table 2. The distributions of AQ scores are typical of normally developing populations. As a general comparison, the mean total AQ of fifty-five native speakers of English at a British university in [45] was 102 (SD = 14.5, range = 71–150) and the mean total AQ of sixty native speakers of English at an American university in [31] was 110.05 (SD = 18, range = 78–155).

**Table 2:** Descriptive statistics of subject-level variables.

Condition		gay	straight
**Positive**		20 subjects, 9F	24 subjects, 13F
	AGE	20.37 (4.39)	20.33 (2.16)
	ATTITUDE	2.80 (1.24)	3.63 (1.395)
	TOTAL AUTISM-SPECTRUM QUOTIENT	113 (13.85)	104.17 (15.88)
	SOCIAL SKILLS	21.60 (4.92)	20.38 (5.55)
	ATTENTION SWITCHING	25.65 (3.50)	23.96 (3.77)
	ATTENTION TO DETAIL	25.60 (5.98)	23.42 (5.40)
	COMMUNICATION SKILLS	21.30 (2.99)	18.46 (3.95)
	IMAGINATION	18.85 (4.63)	17.96 (3.67)
	BIG FIVE INVENTORY		
	EXTROVERSION	3.06 (0.77)	3.03 (0.73)
	AGREEABLENESS	3.41 (0.68)	3.67 (0.52)
	CONSCIENTIOUSNESS	3.04 (0.71)	3.38 (0.60)
	NEUROTICISM	2.77 (0.85)	2.96 (0.84)
	OPENNESS	3.90 (0.60)	3.84 (0.46)
	RSPAN	64.65 (7.03)	61.96 (9.82)
	Pre-exposure VOT (MS)	80.91 (16.57)	83.78 (14.80)
	Post-exposure VOT (MS)	79.79 (17.62)	82.25 (16.54)
**Negative**		19 subjects, 10F	21 subjects, 13F
	AGE	19.26 (1.19)	20.24 (2.98)
	ATTITUDE	3.32 (1.63)	4.05 (1.563)
	TOTAL AUTISM-SPECTRUM QUOTIENT	108.30 (10.09)	109.90 (13.69)
	SOCIAL SKILLS	20.16 (4.71)	20.19 (4.35)
	ATTENTION SWITCHING	24.84 (3.72)	24.81 (2.91)
	ATTENTION TO DETAILS	25.63 (4.70)	25.48 (3.92)
	COMMUNICATION SKILLS	19.58 (3.72)	20.19 (3.31)
	IMAGINATION	18.05 (2.86)	19.24 (5.12)
	BIG FIVE INVENTORY		
	EXTROVERSION	3.28 (0.81)	3.08 (0.70)
	AGREEABLENESS	3.65 (0.68)	3.43 (0.72)
	CONSCIENTIOUSNESS	3.26 (0.90)	3.16 (0.67)
	NEUROTICISM	2.74 (0.82)	3.07 (0.79)
	OPENNESS	3.70 (0.62)	3.86 (0.57)
	RSPAN	63.11 (8.21)	63.9 (11.09)
	Pre-exposure VOT (MS)	86.72 (13.29)	84.00 (14.83)
	Post-exposure VOT (MS)	86.49 (13.44)	84.45 (15.20)

Mean and standard deviation of variables measured for subjects in each narrative condition, including age, attitude towards the narrator, total AQ, AQ subscores, Big 5 subscores, and RSPAN, as well as the VOT values during the pre- and post-exposure blocks.

## Analysis

We are interested in two questions about subjects’ VOT productions. First, how does VOT shift as a result of hearing the narrative, across subjects, after controlling for other factors? Second, what factors affect how much a subject’s VOT shifts?

Our analysis addresses these questions using a two-step modeling procedure. We first model the effects of properties of all factors on VOT *except* whether the subject has heard the narrative yet or not (Model 1). The residuals of this model are VOT values normalized for speaking rate, properties of the host word, and idiosyncratic by-subject and by-word differences. For each word for each subject, we then calculate the *normalized VOT shift* (just *shift* henceforth): the difference between the subject’s pre-narrative and post-narrative normalized VOT values for the word. We then model the effects of subject-level variables (such as rspan and attitude towards the narrator) on the amount of shift (Model 2). The results of Model 2 address both questions: the value of its intercept corresponds to how much overall VOT shift occurs, and the values of its coefficients describe how different subject-level variables affect the amount of shift.

### Model Preliminaries

The models include several types of predictors, summarized in Table 3, corresponding to properties of the host word (word-level predictors), the individual utterance (utterance-level predictors), the narrative condition (outcome, sexuality), or the subject (all other predictors). For the purposes of modeling it is convenient to call all predictors indexing either properties of the narrative or of the subject “subject-level”, meaning that they have fixed values for all tokens from a given subject.

**Table 3:** Predictors used in Models 1 and 2.

Predictor type	Predictor	Abbreviation	Type
*Word-level*	Number of syllables	syllables	ordered factor (1, 2, 3, 4)
	Log celex frequency	frequency	continuous
	Initial consonant	consonant	factor (/p/,/t/,/k/)
*Utterance-level*	Syllables/second in the word	rate1	continuous
	Syllables/second in the carrier phrase	rate2	continuous
	Within-block order	trial	continuous
	Stimulus block	block	factor (pre-, post-narrative)
*Subject-level*	Narrative outcome	outcome	factor (positive, negative)
	Narrator’s sexual orientation	sexuality	factor (gay, straight)
	Subject gender	gender	factor (male, female)
	Subject attitude	attitude	continuous
	RSPAN score	rspan	continuous
	Openness	o	continuous
	Conscientiousness	c	continuous
	Extraversion	e	continuous
	Neuroticism	n	continuous
	Agreeableness	a	continuous
	Attention switching	as	continuous
	Social skills	ss	continuous
	Communication	cm	continuous
	Imagination	im	continuous
	Attention to detail	ad	continuous

Model 1 describes how VOT depends on word-level and utterance-level predictors, with the exception of block : the frequency, initial consonant, and length in syllables of the host word, as well as two measures of speaking rate and the within-block position of the utterance. Speaking rate (syllables per second) within the host word (rate1) and within the carrier phrase (rate2) were calculated using word boundaries from forced alignment obtained using the Penn Forced Aligner (http://www.ling.upenn.edu/phonetics/p2fa/; [65]) with the number of syllables in a word or carrier phrase determined assuming canonical American English pronunciations from the CMU pronunciation dictionary (http://www.speech.cs.cmu.edu/cgi-bin/cmudict). To ensure that each force-aligned chunk corresponded to the number of syllables indicated by its transcription, all disfluencies (such as false starts, repetitions, and non-speech noises) which deviated from the prompts were segmented out by hand prior to forced alignment. All resulting force-aligned word and phrase boundaries were very good in a visual inspection of several speakers’ files; as a result, we were confident in the results of forced alignment, and made no explicit comparison with manually-labeled boundaries.

Model 2 describes how the amount of normalized VOT shift depends on subject-level predictors: the narrative outcome and the narrator’s sexual orientation, as well as the subject’s gender, attitude towards the narrator, RSPAN, Big 5 personality scores (c, o, e, a, n) and AQ subscores (ss, cs, im, ad, as).

### Model 1

VOT in the dataset was modeled using a linear mixed-effects model fit in R, using the lmer() function from the lme4 package [66].

Fixed-effect terms were included for frequency, consonant, and syllables, to allow for the possibility that VOT is negatively correlated with frequency, to control for the well-known effect of place of articulation on VOT (/p/</t/</k/; e.g., [67]), and to account for a trend observed in our data for VOT to depend on the number of syllables in the word. Fixed-effect terms were also included for rate1 and rate2, to control for the large negative effects of speaking rate on VOT (e.g., [68], [69]). By-subject random slopes were included for all five variables, to allow for by-subject variability in the effect of each variable on VOT [63], [70].

Exploratory data analysis suggested that some subjects’ VOT values steadily increased or decreased over the course of a block (pre or post-narrative), and that the slope of this change could differ by block. To control for this possibility, we included both fixed-effect terms and random slopes for the main effect of trial and for its interaction with block.

The model also included by-subject and by-word random intercepts, to allow for subject-specific variation in VOT [61], as well as word-specific variation in VOT beyond the effects of the word-level predictors. Finally, the model included all possible correlations between random effect terms. The model formula in lme4 style was: vot ∼ consonant+frequency+syllables+rate1+ rate2+ trial+trial : block+(1+ consonant+frequency+syllables+rate1+ rate2+ trial+trial : block 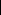 subject)+(1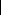 word). By-word random slopes for utterance-level predictors could also be included for the maximal random effect structure, following [71]. A model with by-word random slopes added failed to converge, and was extremely similar to the model without them; the latter is reported for simplicity.

Outliers were trimmed from the dataset prior to fitting the model. A token for a given word and subject was excluded if its VOT was more than 3 standard deviations from the mean across all tokens of the word, all tokens from the subject, or the entire dataset. Because of the extremely strong effect of speaking rate on VOT, we also excluded speaking rate outliers (either rate1 or rate2) by the same criterion. Out of 11573 tokens in the dataset described above, we excluded 134 tokens (1.1%) as VOT outliers and 216 tokens (1.9%) as rate outliers. To reduce multicollinearity between predictors, rate1, rate2, and trial were standardized (centered and divided by one standard deviation), block was sum-coded, consonant was Helmert-coded, and syllables was treated as an ordered factor using orthogonal polynomial coding. syllables was treated as a factor rather than a continuous predictor because of its small number of unique values (4). Exploratory plots suggested the relationship between syllables and VOT was roughly quadratic, so only the linear and quadratic trend terms (and not the cubic term) for syllables were included.

The residuals of an initial fit of the model had a distribution which deviated strongly from normality. We trimmed 80 tokens (0.7%) with residuals which were more than 3 standard deviations from the mean, and refit the model to the trimmed dataset. The new model had a residual distribution much closer to normality, and it is the residuals of the new model which were used as the input to Model 2.

#### Model 1: Results

Table 4 lists the estimated value for each fixed-effect coefficient, along with its standard error, 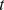 statistic, and corresponding significance value using a Wald test. Each coefficient’s 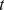 statistic should be normally distributed given the size of the current dataset, making a Wald test appropriate [72]. Because all predictors have been centered, the intercept (86.3 msec) can be interpreted as the predicted mean VOT across the three places of articulations, for a word with average values of each other word-level predictor, for an average subject.

**Table 4:** Model 1 summary.

Predictor	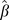	s.e.( 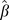 )	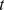	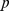
Intercept	86.31	1.93	44.61	<0.0001
Consonant (t vs. p)	10.41	1.28	8.13	<0.0001
Initial consonant (k vs. p/t)	3.10	0.70	4.40	<0.0001
Log celex frequency	1.24	1.12	1.11	0.27
Number of syllables (linear)	17.99	3.15	5.71	<0.0001
Number of syllables (quadratic)	6.93	2.39	2.91	0.0037
Syllables/second (word)	−9.77	0.69	−14.21	<0.0001
Syllables/second (phrase)	−3.58	0.72	−5.00	<0.0001
Within-block order	0.53	0.23	2.35	0.019
Within-block order×stimulus block	0.07	0.48	0.14	0.89

Estimate (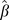), standard error (s.e.(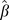)), 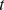-value, and significance value (Wald test) for each fixed-effect coefficient in Model 1.

**Figure 1:**
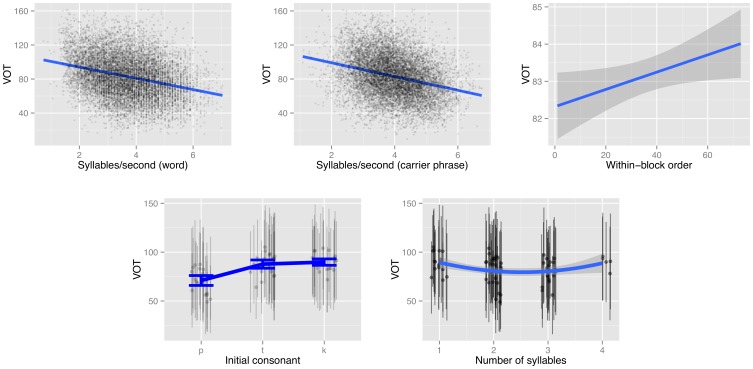
Empirical plots of VOT versus significant predictors in Model 1. For the utterance-level predictors rate1, rate2, and trial (top row), the line and shading show a linear fit and 95% confidence intervals (CIs) to the empirical data, represented by one point per observation (points omitted in the trial plot for legibility). For the word-level predictors consonant (bottom left) and syllables (bottom right), each point and vertical line show the mean and its 95% CI for VOT over all tokens of one word. The error bars for consonant are 95% CIs on the mean of the word-level means; the curve and shading show a quadratic fit to the word-level means and its 95% CIs, corresponding to the coding of syllables (see text).

Most word-level predictors have significant effects on VOT. Figure 1 illustrates the empirical relationships between the significant predictors and VOT. As shown in the lower-left panel of Figure 1, VOT is heavily modulated by the place of articulation of the consonant. The model predicts that the VOT of velars is 9.30 msec longer than the VOT of more anterior stops (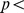 0.0001) while the VOT for coronals is 20.82 msec longer than for labials (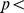 0.0001). Because the interpretation of the two Helmert contrasts for consonant is “half the difference between/t/and/p/” and “one third the difference between/k/and the mean of/p/and/t/”, i.e., 9.30 msec = 3.3.10. As illustrated by the lower-right panel of Figure 1, VOT depends on word length (in syllables: linear trend 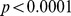, quadratic trend 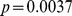), with predicted VOT of 77.7/78.8/86.9/101.8 msec for words of 1/2/3/4 syllables. The upper-left two panels of Figure 1 show that VOT is strongly negatively affected by both measures of speaking rate (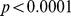): the model predicts a decrease of 9.77 msec per increase of one standard deviation (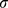) in syllables/second within the host word, and a decrease of 3.58 msec per 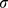 increase in syllables/second within the carrier phrase. The host word’s within-block order, as illustrated by the top-right panel of Figure 1, also has a small but significant effect on VOT (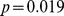), which is predicted to increase by roughly 2.12 msec over the course of each block (corresponding to a 4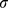 increase in trial); this effect does not differ significantly between the pre- and post-narrative blocks (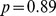). The effect of log frequency did not reach significance (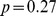).

The by-subject and by-word random intercept variances have estimated values 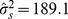 and 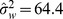, and are both highly significant (i.e., 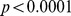 using a likelihood ratio test [72]). Thus, subjects and words differ in their average VOT, *after* controlling for all word-level and utterance-level predictors (except for block): 95% of words are predicted to have offsets of less than 16 msec (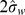) and 95% of subjects are predicted to have offsets of less than 28 msec (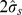), relative to the grand mean.

### Model 2

The residuals of Model 1 give a measure of normalized VOT for each token, after controlling for word-level and utterance-level factors (except for block). The difference in normalized VOT between the post-narrative and pre-narrative blocks, or *normalized VOT shift*, measures how much a subject shifted her VOT for a particular word as a result of hearing the narrative.

Normalized VOT shift was again modeled using a linear mixed-effects model, using the lmer() function in lme4. To determine the effect of properties of the narrative and the subject on the amount of shift, a fixed-effect term was included for each subject-level predictor (Table 3). By-word random intercepts were included to allow for word-specific variation in the amount of shift, and by-subject random intercepts were included to allow for subject-specific variation in the amount of shift, beyond the effects of subject-level predictors. The model formula in lme4 style was: shift ∼ gender + attitude + sexuality + outcome + rspan + o + c + e + n + a + as + ss + cm + im + ad + (1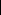 subject) + (1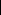 word). As for Model 1 (see note ??), the maximal model would incorporate by-word random slopes. (By-speaker random slopes are not possible since the model contains only speaker-level predictors.) A model with by-word random slopes added was not significantly different (using a likelihood ratio test) to the model without them; the latter is reported for simplicity.

Due to the data trimming steps taken in Model 1, as well as the exclusion of some tokens from the full dataset (see above), in 231 cases either the pre-narrative or post-narrative token for a given subject and word was not assigned a normalized VOT, in which case the normalized VOT shift was undefined. Model 2 was fit to the 5348 tokens for which normalized VOT shift was defined, each corresponding to one of 70 words for one of 84 subjects.

To reduce multicollinearity between predictors, continuous predictors (attitude, rspan, Big 5 scores, AQ subscores) were centered, and gender, sexuality, and outcome were sum-coded (e.g., for outcome : positive = 0.5, negative = −0.5). Each continuous predictor was scaled by twice its standard deviation, in order to make the fixed-effect coefficients for continuous predictors comparable to those for categorical predictors [73].

The condition number of the (centered) predictors was 4.8, indicating minimal multicollinearity in the full set of predictors [74]. Nonetheless, some moderate correlations exist among Big 5 scores and AQ subscores, such as between ss and cm (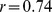).

#### Model 2: Results

Table 5 lists the estimated value for each fixed-effect coefficient, along with its standard error, 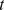-value, and significance values obtained by MCMC sampling from the posterior distribution of the model parameters, with 50000 samples, using mcmcsamp() in lme4. Because all predictors have been centered, the intercept can be interpreted as the predicted amount of normalized VOT shift for an average subject and average word. The predicted amount of shift is very small (0.47 msec) and is not significant (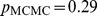), meaning that there is no evidence that VOT is lengthened, on average, as a result of listening to the narrative. However, five predictors did have significant effects on the amount of normalized VOT shift (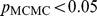), suggesting that individual subjects did shift towards or away from the narrator, in part as a function of some subject-level predictors. One of these predictors (i.e. conscientiousness) was not consistently significant under different model parameterizations and data trimming procedures we tried before arriving at a final model. We will therefore only consider the four other predictors (attitude, outcome, openness, attention-switching) as significantly affecting the amount of normalized VOT shift.

**Table 5:** Model 2 summary.

Predictor	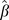	s.e.( 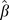 )	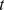	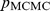
Intercept	0.47	0.47	1.00	0.30
Subject gender	0.94	0.99	0.95	0.33
Subject attitude	−4.53	1.02	−4.44	<0.0001
Narrator sexual orientation	1.63	1.06	1.54	0.11
Narrative outcome	−2.58	0.96	−2.69	0.0082
RSPAN	1.36	1.02	1.33	0.17
Openness	3.53	1.05	3.36	0.0010
Conscientiousness	−2.13	1.11	−1.92	0.043
Extraversion	0.24	1.38	0.17	0.84
Neuroticism	−1.47	1.20	−1.23	0.21
Agreeableness	−2.06	1.11	−1.86	0.059
Attention switching	2.58	1.10	2.35	0.014
Social skills	−2.02	1.87	−0.01	0.99
Communication	−1.29	1.56	−0.83	0.39
Imagination	1.29	1.19	1.08	0.26
Attention to detail	0.07	1.11	0.06	0.95

Estimate (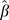), standard error (s.e.(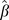)), 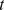-value, and simulation-based significance value for each fixed-effect coefficient in Model 2.

**Figure 2:**
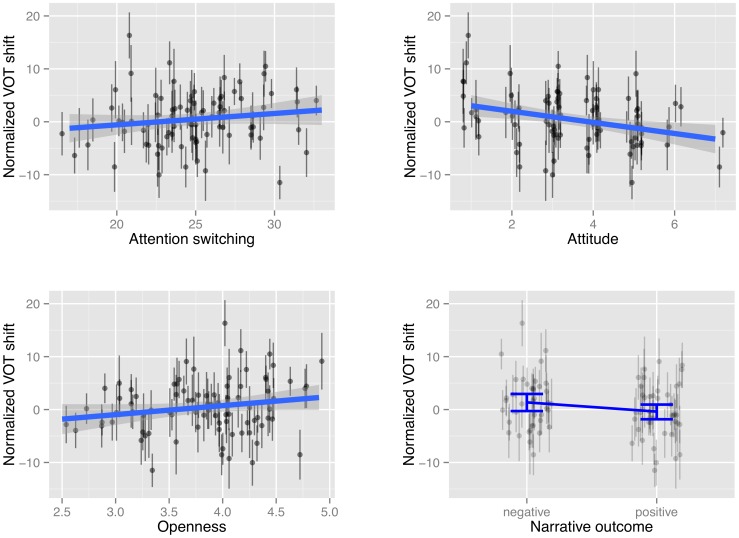
Empirical plots of normalized VOT shift versus significant by-subject predictors in Model 2. Each point and vertical line show the mean and its 95% confidence interval for one subject’s shift across all words. For as (top left), attitude (top right), and o (bottom left), lines and shading show a linear fit and 95% CIs of normalized VOT shift vs. the predictor, across all tokens. For outcome (bottom right), the error bars are 95% CIs on the mean of normalized VOT shift across all tokens.

Figure 2 illustrates the effects of these predictors on the amount of shift. The effects of attitude and openness are highly significant. As shown in the top-right panel of Figure 2, subjects with a more positive attitude towards the narrator (lower attitude score) shift towards him (increased VOT) while those with a negative attitude shift away from him (decreased VOT) (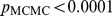), with an increase of 2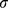 in attitude corresponding to a decrease of 4.53 msec in predicted VOT shift (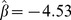). The lower-left panel of Figure 2 illustrates that subjects with higher Openness scores increased VOT, while those with lower Openness scores decreased VOT (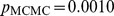), with an increase of 2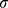 in Openness corresponding to an increase of 3.53 msec in predicted VOT shift. The lower-right panel of Figure 2 shows that the amount of VOT shift differed by 2.58 msec between subjects in the negative and positive outcome conditions (negative condition>positive condition; 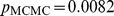). There was also a significant effect of Attention Switching (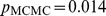). As shown in the top-left panel of Figure 2, an increase of 2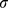 in as score is predicted to increase VOT shift by 2.58 msec. No other predictor, including subject gender and perceived narrator sexual orientation, had a significant effect.

The by-word random intercept has estimated value of 0, meaning there is no evidence that words differed in the amount of VOT shift. The by-subject random intercept variance has an estimated value of 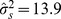, and is highly significant (i.e., 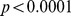 using a likelihood ratio test [72]. Thus, subjects differ in how much they shift VOT, *after* controlling for all subject-level predictors, with 95% of subjects expected to shift VOT between −6.83 msec and 7.77 msec ( = 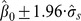, where 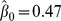 is the predicted value of the intercept), beyond the effect of subject-level predictors included in the model. These by-subject offsets could be due to systematic variability between subjects in the amount of VOT shift (i.e., effects of subject-level predictors which were not included in the model), truly idiosyncratic variability among subjects in the amount of VOT shift, or a combination of the two. Thus, although subjects do not show any VOT shift *on average*, there are substantial differences in the amount of VOT shift shown by different subjects, due in part to characteristics of subjects and the narrative.

A natural question is how important the full set of subject-level predictors is for predicting the amount of VOT shift. One metric of the importance of a set of predictors for linear mixed models is the reduction in mean-squared prediction error of a full model which includes these predictors, relative to a baseline model which does not [75]; this quantity, denoted 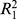, lies between 0 and 1. In our case, this measure is 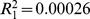 relative to a baseline model with only by-word and by-subject random intercepts, indicating that while the subject-level predictors make significant contributions to the model of VOT shift, they explain very little of the total observed variability in VOT shift.

Also of interest is the relative importance of the different predictors in the model. Because the predictors were standardized, the coefficient values are comparable, and one measure of a predictor’s importance is simply the absolute value of its coefficient (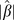). Another measure of a predictor’s importance is the percentage change in 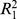 when the predictor is dropped from the full model. Both measures are shown in Table 6, with predictors sorted in order of 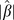. The four significant predictors in Model 2 have the same ordering under both measures, and come out as more important than other predictors by both measures. (For other predictors the two measures of importance disagree somewhat.).

**Table 6:** Relative importance of predictors in Model 2.

Predictor	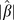	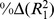
Subject attitude	4.53	65.6
Openness	3.53	36.6
Narrative outcome	2.58	22.1
Attention switching	2.58	16.0
Conscientiousness	2.13	9.3
Agreeableness	2.06	8.6
Narrator sexual orientation	1.63	4.7
Neuroticism	1.47	2.1
RSPAN	1.36	2.8
Communication	1.29	−1.4
Imagination	1.29	0.6
Subject gender	0.94	−0.3
Extraversion	0.24	−3.5
Attention to detail	0.07	−3.6
Social skills	0.02	−3.6

Absolute value of the fixed effect coefficient (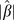) for each predictor, and percent change in 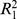 when it is dropped from the model.

#### Comparison to one-step model

Note that instead of the two-step modeling procedure used here, it is also possible to address both questions of interest (how much overall VOT shift occurs, and which subject-level variables affect the amount of shift) using a single more complex model, with terms *both* corresponding to the amount of VOT shift (as in Model 2), and controls for other factors which affect VOT (as in Model 1). In such a `one-step’ model, the main effect for block would correspond to an overall shift in VOT (the intercept in Model 2), and interactions of block with subject-level predictors would correspond to factors which affect the amount of VOT shift (the main effects in Model 2). A pilot version of this study [76] used a one-step modeling procedure. We have used the two-step procedure here for ease of presentation, since it has allowed us to focus our discussion on the results of Model 2 in detail. However, we note that fitting a one-step model to the current dataset yields broadly similar results to Model 2, with respect to how much shift occurs and which factors affect the amount of shift. In the one-step model, as in Model 2, there is not a significant overall shift in VOT, and there are significant effects of outcome, o, attitude, and as on the amount of shift, all in the same directions as in Model 2. However, in the one-step model two additional subject-level predictors significantly affect the amount of shift: agreeableness and narrator sexual orientation, both predictors which were near at least marginal significance (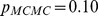) in Model 2. One possible explanation for this discrepancy is that the two-step model has less statistical power than the one-step model to detect factors affecting the amount of VOT shift, since it is based on less data: one observation per subject/word pair instead of two, and more points discarded as a result of trimming outliers. In any event, while further investigation is needed to ascertain the significance of these effects, the potential significance of agreeableness and narrator sexual orientation suggest that individuals who are less agreeable and those who participated in the gay narrator condition tend to show a positive VOT shift.

## General Discussion

Our findings show that phonetic imitation is highly variable, both in terms of contexts and across individuals. Before discussing the implications of such findings, however, it is worth noting that the significance of these findings is partly contingent on understanding that any observed VOT shift between production blocks *is* in fact due to exposure to the narrative with extended VOT. To be sure, since the design of this study did not include a separate condition where the VOTs were unchanged in the narrative, one might question whether the observed VOT shifts were imitations at all. That is, would the VOT shifts take place even if the model talker’s VOTs had not been extended? For example, subjects with higher o scores (openness to new experiences) might have increased their VOTs from the first to the second reading of the word list more than subjects with lower o scores, regardless of whether there were an intervening narrative. While we acknowledge the possibility of such an interpretation, we believe that this alternative interpration is difficult to reconcile with the general findings of this study. To begin with, we found no *unmediated* VOT shifts overall, suggesting that the participants did not change their VOTs between blocks in general. To the extend that VOT shifts are observed, how much a subject’s VOT shifted is predicted in part by subject-level predictors, some of which crucially referenced the presence of the narrative. For example, the amount of VOT shift is determined, if only partially, by subjects’ attitude toward the narrator. These results point to a significant awareness on the part of the participants of the content of the narrative. The direction of VOT shifts is also nonrandom; subjects with a positive attitude towards the narrator increase VOT more than subjects with a negative attitude towards the narrator. If we did not assume subjects’ VOTs shifted as a result of exposure to the narrative, we would be forced to conclude that the strong effect of attitude on the amount of VOT shift (subjects who like the narrator more shift towards him more) is actually spurious: subjects who liked the narrator more tended to be those who increase VOT more, purely by chance. Given these reasons, we assume for the remainder of this discussion that between-subject differences in the amount of VOT shift reflect between-subject differences in the effect of the narrative on their speech, in line with previous studies of phonetic imitation, which mostly have not used a control condition. However, future work using a control condition should test the assumption implicit in much of the phonetic imitation literature, that effects of covariates on how subjects imitate a speech stimulus are in fact due to exposure to the stimulus.

With the above caveat in place, our findings point to the fact that phonetic imitation, defined here as shifts in VOT between production blocks, may be modulated by disincentives and obstacles that conflict with goals, attention, and liking [1]. To begin with, in line with previous literature on phonetic convergence and imitation, which showed significant social modulation of imitation effects (e.g., conversational role [14], [16], national identity [27], race/ethnicity [7]), the present study also found that the extent of phonetic imitation crucially depends on what impression the listener has toward the person who produces the phonetic variant. In particular, subjects who liked the narrator more imitated his extended VOT more. Relative to other predictors considered in Model 2, this attitudinal measure is also one of a small number of predictors that significantly influence the amount of phonetic imitation, highlighting the prominent role “liking” has in mediating the perception-production linkage.

Our study also found that the extent of phonetic imitation depends on where the phonetic variant is embedded (i.e. the content of the narrative), even if the effect of narrative outcome on imitation might seem surprising at first glance. Recall that there were two possible outcomes to the blind date as recounted by the narrator during the *listening* phase of the experiment. In the positive scenario, the narrator and his date went on well, while in the negative scenario, the narrator behaved rudely by leaving the blind date in a lurch. Perhaps counterintuitively, subjects who heard the negative narrative show an increase in VOT in the post-exposure block. To be sure, there is no correlation between subject’s attitude toward the narrator and the narrative outcome (i.e., 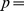
0.29 for a Wilcoxson rank-sum test of the hypothesis that attitude differs between the positive and negative narrative conditions). That is, it is not the case that subjects tended to react positively toward the narrator under the negative scenario. Why should subjects imitate more when they heard the negative story compared to those who heard the positive one? One, admittedly speculative, possibility is that subjects who heard the negative story paid more attention to the narrative than those who heard the positive one on account of the fact that the negative story is more engaging than the positive account. This interpretation may be related to the notion of *automatic vigilance*, which has been argued to be a mechanism that serves to direct attentional capacity to undesirable stimuli without the perceiver’s intention or control [77]. If this is the case, it would suggest that subjects’ attention and focus is driving this narrative outcome effect.

We found that the dynamics of VOT imitation were modulated by speaker attitude and narrative outcome, but not speaker gender or perceived sexual orientation of the narrator. The former can be thought of as variables which are constructed situationally, and the latter as “macro” social variables describing pre-existing categories. To the extent that our experiment is representative of phonetic imitation behavior more generally, this asymmetry highlights the importance of taking into account variables which are defined relative to a particular social situation when studying phonetic imitation, in line with other recent work in sociophonetics (e.g., [16], [78], [79]).

Beyond these situation-specific social factors, our findings also show that certain aspects of the social and cognitive makeup of the subject strongly influence the extent of phonetic imitation. In particular, individuals whose personality reflects a greater sense of openness and those with strong attention focus, as measured by the attention switching subscore of the AQ, tend to approximate the narrator’s VOT more than those with the opposite personality and autistic-like traits. The personality facet of Openness (also known as Openness/Intellect) indexes an individual’s level of engagement with perceptual, sensory, as well as abstract and semantic information. The fact that Openness modulates phonetic imitation suggests that the level of engagement with the exposure materials matters. That is, a higher level of engagement with the narrator’s speech may have led to greater attention paid to how the utterances are produced by the narrator. Similarly, individuals who are more focused and are not accustomed to constant attention switching, as indexed by a high attention switching subscore of the AQ, might likewise be more attuned to the fine-grained phonetic fluctuations in the exposure materials, thus increasing the chance of such phonetic attributes being imitated. To be sure, further investigation is required to understand the way selective attention and attentional-resource allocation may influence phonetic imitation.

While certain individual-difference dimensions may influence phonetic imitation, it is also important to point out that not all individual-difference dimensions have such an influence, as shown by the lack of an effect of most of the other AQ subscores and personality traits on the amount of VOT shift. Our finding that the extent of phonetic imitation is in part governed by a personality facet that indexes willingness to engage with new information, and a cognitive processing style that favors focused attention and eschews spreading attentional resources thinly, points to the potential role of attention in mediating the perception-production link. To that end, it came as a surprise that working memory capacity, as measured by RSPAN, did not emerge as a significant predictor in phonetic imitation, given that selective attention is highly influenced by working memory resources. This result suggests that success in phonetic imitation might not be related to the availability of attentional resources *per se*, but more related to the monitoring and allocation of attentional resources; both are within the purview of the executive-function process. Further research is needed to elucidate the role of executive-function ability in phonetic imitation.

The present findings have implications for models of speech perception and production, particularly for exemplar-based models of speech production and perception, which assume some form of perception-production feedback loop where exposure to a production activates similar stored exemplars, leading to a subsequent production by the listener-turned-speaker that is more like that of the model talker. Such models are able to account for imitation results [80]–[82], although the inclusion of an attention-weighting component in the model (see, for example, [81]) is needed to account for attention-related inter-individual variation. Further modification is also needed to take into account situationally-based social information in mediating the perception-production link. As noted in [7], simple automatic exemplar-models that predict cumulative imitation effects as a result of increased activation from increased exposure are not tenable in light of findings like those reported here where exposure to the model talker does not necessarily lead to significant overall imitation.

Given that our experiment focused on VOT as the imitation target and employed a very similar methodology to the experiment reported in Nielsen’s study [10], the fact that we did not observe an overall effect of phonetic imitation but she did deserves some qualification. To begin with, the exposure materials in Nielsen’s study were English words presented in isolation, while our exposure materials were embedded in a meaningful narrative. The marked difference in experimental results might be partly attributable to the decontextualization of the exposure materials in Nielsen’s study; imitation might be more automatic (i.e., they can occur without the speaker’s intention or control) in a context where the words are presented in isolation devoid of social significance. The narrative in the present study, in contrast, allows participants to make evaluative judgements on the narrator as he recounts his blind date. The difference in the presence of baseline imitation could also be related to the substantially different statistical analyses used in Nielsen’s study and in the current study. Differences in the model talkers in our experiment and Nielsen’s also might have contributed to the differences in results, in light of previous work showing that shadowers are more likely to accommodate to particular individuals than to others [20].

Another possibility not directly explored here concerns the subject’s interpretation of the social meaning of the extended VOT. Recent studies on the social meaning of released/t/argue that/t/release is associated with qualities such as being educated, elegance, articulateness, and prissiness [83]. This feature has apparently been recruited by particular social groups in their construction of an articulate persona (e.g., nerd girls [84], Orthodox Jewish boys [85], gay divas [79], United States politicians [86]). The association of released consonants with articulateness is partly confirmed in the results of the post-experiment survey in our study. When asked whether they noticed anything unusual about the narrator’s speech, many subjects characterized his way of speaking as “articulate”, “aspirated”, or “robotic”. Given the complex social meaning associated with the released/t/variable, we cannot discount the possibility that the social meaning of an especially strong consonantal release (lengthened VOT) might have an effect on phonetic convergence or divergence. While the indexical meanings associated with released/t/are not intrinsically positive or negative, some subjects might nonetheless resist extending their VOTs in order to avoid projecting an articulate persona.

## Conclusion

This study offers further evidence that the extent of phonetic imitation is highly regulated by individual-level variables, such as an individual’s evaluative judgement of her interlocutor, and the social and cognitive profile of the individual. In particular, such cross-individual variability strongly affects the likelihood and directionality of phonetic imitation.

## Supporting Information

Supporting Information S1 **The full texts of the heterosexual version of the “positive” and “negative” storylines.**
(PDF)
