# Supplementary material for: Phonetic Imitation from an Individual-Difference Perspective: Subjective Attitude, Personality and “Autistic” Traits
Source: PLoS One. 2013 Sep 30;8(9):e74746. doi: 10.1371/journal.pone.0074746 (PMC3786990; doi:10.1371/journal.pone.0074746)
Supplement: Supporting Information S1 — The full texts of the heterosexual version of the “positive” and “negative” storylines. (PDF) [file pone.0074746.s001.pdf]

# Supporting Information

## Positive story

Hi, my name is Larry. So last night I had an amazing date with this guy/girl I met online. This was my **tenth** stab at finding love through a **personal** ad—I guess I'm just your **typical picky Pisces**. So here's what happened.

**Picture** this: we got a **table** on the **patio** of "**Pearl**", a new restaurant in **town**. I immediately found my date Glen/Glenda, a **chiropractor**, more than **tolerable**. He/She's **kinda tubby** and a bit of a **talker**, but there was something about him/her that really made me **tingle**, let's just **put** it that way. The evening was **cordial** at first, but soon I was fully enthralled by this **taunting** man/woman, as he/she sat **pensively** over a **pink cocktail**, his/her **perfect** face lit up by the **candlelight**. His/Her **kissable** lips and **toffee** eyes were a **total turn-on**. I had never met anyone who was able to **captivate** me like that.

Whenever our eyes made **contact**, there was instant **chemistry**. He/She would look at me **teasingly**, **temptingly**, as if **coaxing** me away from my **corpulent**, **pimpled** exes. Around our second **course**—he/she had the **cod** and **cauliflower** and I had a **piece** of **pork** that grew gradually more **tasteless** as I fell head-over-heels—I grew **terribly** aware of the **pointlessness** of all my former dating experiences. In spite of his/her **coolness**, I had a single **purpose**: letting this guy/girl know how I felt. There was just a **teensy** issue: how would I express my affections **tearlessly** and without **pain**? I decided being **candid** was the most **tactful** route. So with a **terror-stricken** expression on my **pale** face, I simply said, "Glen/Glenda, I love you."

Then I grabbed a **pair** of **peppermints** and aimed for the new object of my affection, **timidly** but with as much **confidence** as I had, my **pulse** racing. It's a good thing I was already a little **tipsy**, which helped me stay **calm**. I leaned in, **two** inches away, **panic-stricken**, on the **cusp** of giving his/her a **compliment** with a **peck** on the cheek, when I managed to glance at Glen/Glenda's face. My formerly **poker-faced** date suddenly had a **tigerish** look in his/her eye, with a **concrete** look of desire.

So that was the beginning of our sweet **courtship**, and after that we headed back to my **pigsty** of a house. Hope I'm not being a **killjoy** by giving a **cop-out** ending, but that's as much as you need to know!

## Negative story

Hi, my name is Larry. So I had the worst date last night with this guy/girl I met online. This was my **tenth** stab at finding love through a **personal** ad—I guess I'm just your **typical picky Pisces**. So here's what happened.

**Picture** this: we got a **table** on the **patio** of "**Pearl**", a new restaurant in **town**. My date Glen/Glenda, a **chiropractor**, was **tolerable** enough but a bit of a **talker**, and frankly **kinda corpulent**. He/she didn't exactly make me **tingle**, let's **put** it that way. Around our second **course -** he/she had the **cod** and **cauliflower** and I had a **tasteless piece** of **pork -** I grew **terribly** aware of the **pointlessness** of this dinner date. I was just on the verge of making up some excuse that would get me out of this overly **cordial** dinner, when I saw him/her.

This **taunting** man/woman was sitting **pensively** over a **pink cocktail**, his/her **perfect** face light up by the **candlelight**. His/her **kissable** lips and **toffee** eyes were a **total turn-on**. I had never met anyone who was able to **captivate** me like that.

When our eyes made **contact** there was instant **chemistry**. He/she looked at me **teasingly**, **temptingly**, as if **coaxing** me away from my **pimpled** friend. I had a single **purpose**: getting this guy's/girl's phone number. There was just a **teensy** issue: how do I get rid of my date **tearlessly**. I decided being **candid** was the most **tactful** route. So I simply said, 'thank you, but I don't think it would work out', and got up, leaving **tubby** Glen/Glenda behind with a **terror-stricken** expression on his/her **pale** face.

I grabbed a **pair** of **peppermints** and aimed for the new object of my affection **timidly** but with as much **confidence** and **coolness** as I had, my **pulse** racing. It's a good thing I was already a little **tipsy**, which helped me stay **calm**.

There I stood **two** feet away, **panic-stricken**, visibly in **pain**, on the **cusp** of opening my mouth to mumble some **cop-out compliment**, when there and then his/her **poker-faced** date got back from the bathroom and gave my **tigerish** mystery man/woman a **peck** on the cheek. Well that certainly threw a wrench in my not-so-**concrete** strategy. Man what a **killjoy**!

So that was the end of our short but sweet **courtship** and all there was left for me then was getting back to my **pigsty** of a house and hitting the sack—alone.
